# An open architecture for the massively parallel emulation of the Drosophila brain on multiple GPUs

**DOI:** 10.1186/1471-2202-13-S1-P99

**Published:** 2012-07-16

**Authors:** Lev E Givon, Aurel A Lazar

**Affiliations:** 1 Department of Electrical Engineering, Columbia University, New York, NY 10027, USA

## 

The fruit fly Drosophila melanogaster is an exceedingly useful model organism for studying the causal links between neural circuits and behavior due to the numerical tractability of its brain and its powerful neurogenetic toolkit. Recent progress made in identifying the connectome of the fruit fly [1,2] and in characterizing the input and output functions of its sensory neural circuits [3] raise the possibility of creating and emulating a functional model of the entire fly brain using the increasingly powerful commodity parallel computing technology available to computational neuroscientists. To this end, we have developed an open software architecture for emulating neural circuit modules in the fly brain and their responses to recorded or simulated input stimuli on multiple Graphics Processing Units (GPUs). A key feature of this architecture is its support for integrating instances of different neural circuit models developed by independent researchers by requiring that the models’ implementations provide interoperable interfaces that adhere to the specification prescribed by the architecture.

**Figure 1:**
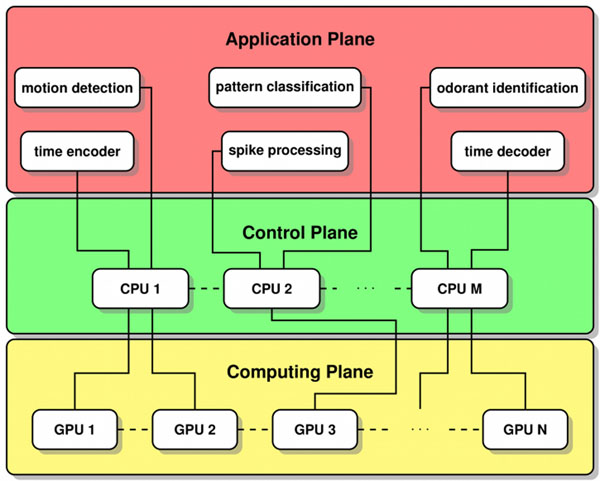
Neurokernel architecture

We refer to the architecture as a Neurokernel because it provides object classes essential to the emulation of the entire fruit fly brain that are analogous to those provided by an operating system kernel: (1) it serves as an extended machine that provides access to neural circuit primitives needed to construct and interconnect models of neural circuit modules in the fly brain; and (2) it serves as a resource allocator that scalably and transparently assigns GPU resources to emulated neural circuit models without manual specification by the researcher [5]. In order to provide these features, the Neurokernel architecture comprises several planes of abstraction that separate its application, control, and computing aspects (Fig. 1). Models of brain function implemented using the architecture use the application plane’s API to access neural circuit primitives without directly specifying which GPU resources to use. The architecture’s control plane automatically partitions and maps circuits to available GPU resources, and manages communication between multiple GPUs hosted locally or remotely. Storage methods used to efficiently represent large networks of neurons and synapses with feedback connections in GPU memory and numerical methods used to update neuron and synapse states are implemented in the computing plane.

We implemented key elements of the Neurokernel software using the Python programming language and the PyCUDA interface to NVIDIA’s CUDA GPU programming environment [4] to avail ourselves of the increasingly powerful ecosystem of scientific computing Python software and make the architecture accessible to other researchers in the neuroscience community.
